# Ureteroiliac artery fistula caused by full-length metallic ureteral stenting in a malignant ureteral obstruction: a case report

**DOI:** 10.1186/s13256-020-02532-4

**Published:** 2020-10-19

**Authors:** Yasuyuki Miyauchi, Yu Osaki, Hirohito Naito, Hiroyuki Tsunemori, Megumi Itoh, Kenji Kanenishi, Takashi Norikane, Takayuki Sanomura, Yoshihiro Nishiyama, Mikio Sugimoto

**Affiliations:** 1 Department of Urology, Kagawa University Faculty of Medicine, 1750-1 Ikenobe, Miki-cho. Kita-gun, Kagawa, 761-0793 Japan; 2 Department of Perinatology and Gynecology, Kagawa University Faculty of Medicine, Kagawa, Japan; 3 Department of Radiology, Kagawa University Hospital, Kagawa, Japan

**Keywords:** Metal stent, Metallic ureteral stent, Resonance, Malignant ureteral obstruction, Ureteroarterial fistula, Arterioureteral fistula

## Abstract

**Background:**

The metallic stent is a new device for relieving the urinary tract in patients with malignant ureteral obstruction with short life expectancy and has been used frequently worldwide for its efficacy and safety. A ureteroarterial fistula with indwelling ureteral stent is rare but highly fatal, and there are several reports of ureteroarterial fistula treated by conventional polymer stents, although there are no reports on metallic stents. To our knowledge, this paper describes the first case of a ureteroiliac artery fistula caused by a full-length metallic ureteral stent in malignant ureteral obstruction.

**Case presentation:**

Our patient was a 57-year-old Asian woman with a history of locally advanced cervical cancer who underwent abdominal total hysterectomy and chemoradiotherapy. She was diagnosed with right hydronephrosis and hydroureter secondary to upper ureteral obstruction because of retroperitoneal lymph node metastasis. A urinary tract obstruction after placement of 12 months of polymer stent followed by 18 months of metallic stent was relieved, consequently resulting in intermittent gross hematuria with bladder tamponade and anemia. Contrast-enhanced computed tomography could not reveal a ureteroarterial fistula; however, retrograde pyelography emphasized the existence of a ureteroiliac artery fistula. The patient underwent successful endovascular heparin-bonded stent graft placement, and her gross hematuria disappeared thereafter.

**Conclusion:**

The metallic stent is a useful device for patients with malignant ureteral obstruction with a short life expectancy, although it may impose a higher pressure on the extraureteral tissue than conventional polymer stents due to its properties and may cause a ureteroarterial fistula. The narrowing of the external iliac artery diameter visualized by computed tomography may be helpful for predicting ureteroarterial fistulas.

## Background

A malignant ureteral obstruction (MUO) can be caused by the direct invasion, lymph node metastasis, and peritoneal dissemination of gastrointestinal or gynecologic cancer that compresses the ureter externally [1]. Although MUO is a poor prognostic factor with a median life expectancy of < 1 year [1–5], urologists have increasingly been required to intervene in MUO management in the early stages to improve renal function because of the need for aggressive treatment, including chemotherapy, for the primary disease. To relieve a urinary tract obstruction, the retrograde placement of a polymeric ureteral stent or creation of a nephrostomy have been undertaken conventionally. Because polymeric stents are vulnerable against external pressure, they require replacement every 1–3 months and eventually lose effect. A nephrostomy affects the patient’s appearance and quality of life, and it requires replacement. A full-length metallic stent (Resonance; Cook Medical, Bloomington, IN, USA) has been developed to overcome disadvantages of these conventional approaches and is widely used globally. Several studies have reported the higher patency and success rate by its strong resistance to extrinsic compression [6] and its ability to stay in place for 12 months, thereby eliminating the need for stent replacement in patients with MUO [3, 5]. Moreover, these stents are reportedly safe for use and have lesser complications than ordinary polymer ureteral stents [7–9].

However, metallic stenting can nevertheless have potential complications. Although ureteroarterial fistula is a rare event accompanied by life-threatening complications, there are no reports on full-length metallic stent placement. We present the first case, to our knowledge, of ureteroiliac artery fistula caused by a full-length metallic ureteral stent in a patient with MUO.

## Case presentation

**Fig. 1:**
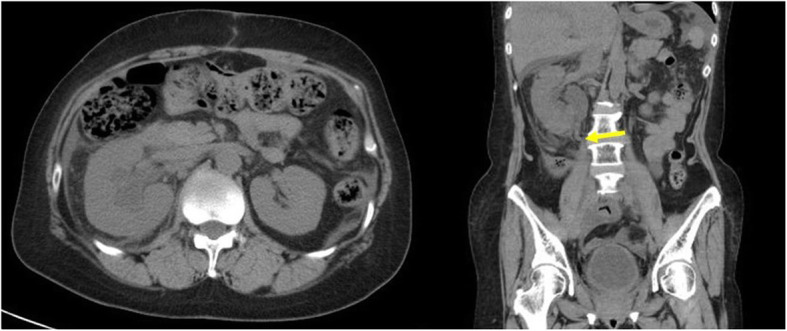
Computed tomography showed a right hydronephrosis in the transverse plane, and the coronal view showed upper ureteral obstruction (arrow)

**Fig. 2:**
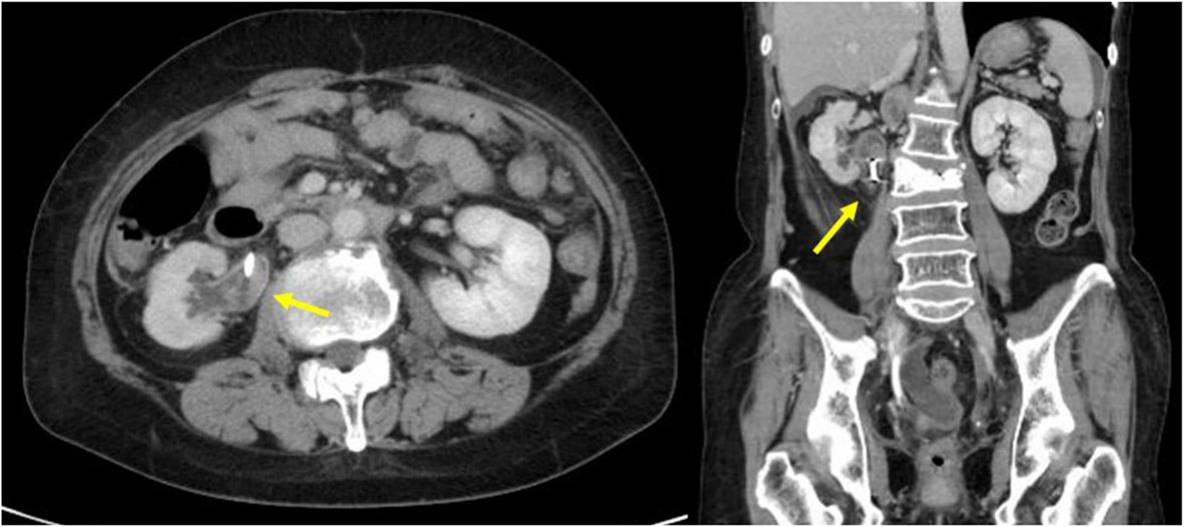
Contrast enhanced computed tomography showed a hematoma at the right ureteropelvic junction (arrows) but did not reveal a bleeding source

**Fig. 3:**
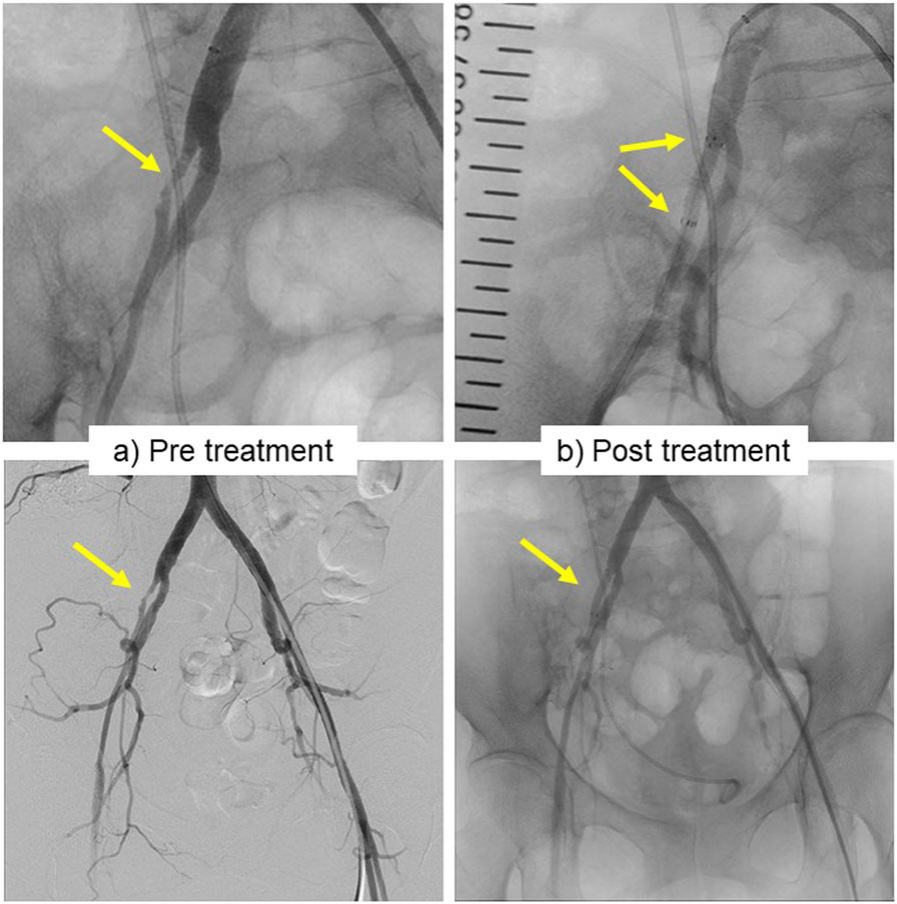
Pretreatment angiography showed narrowing of the external iliac artery (arrows) but did not detect a fistula to the urinary tract (**a**). After expanding the narrowed external iliac artery, a heparinbonded stent-graft was placed (arrows) (**b**)

Our patient was a 57-year-old Asian woman diagnosed with locally advanced cervical cancer (T4aNxM0; stage IVa) 6 years prior to presentation. She had undergone six courses of chemotherapy with paclitaxel and carboplatin, as well as whole pelvic irradiation (57.8 Gy) followed by abdominal total hysterectomy and bilateral salpingo-oophorectomy. However, she developed tumor recurrence at the para-aortic lymph nodes 3 years later and underwent three courses of chemotherapy with paclitaxel and carboplatin again, followed by para-aortic and pelvic lymphadenectomy and omentectomy. Despite the aggressive treatment of her disease, she presented with a new onset of right hydronephrosis and hydroureter secondary to upper ureteral obstruction caused by retroperitoneal lymph node metastasis (Fig. 1). She had complicated pyelonephritis due to MUO; thus, she was referred to our department for securing of the urinary tract and drainage. A double-J polymeric stent was inserted and exchanged at regular intervals every 8 weeks for 1 year. Because of the progression of the disease and the patient’s complaint of frequent stent replacement, we replaced the double-J polymeric stent with a full-length metallic ureteral stent (Resonance). One year after the start of metallic ureteral stenting, we had to replace the ureteral stent because of febrile urinary tract infection. Six months after the second metallic stent placement, the patient complained of gross hematuria that presented bladder tamponade. Cystoscopy was performed by removing the hematoma, although the inside of the bladder was intact. Contrast-enhanced computed tomography (CT) showed a hematoma at the right ureteropelvic junction but did not reveal the bleeding source (Fig. 2). After that, the patient’s intermittent macrohematuria and marked anemia progressed; retrograde pyelography was performed for suspected ureteroiliac artery fistula. When the tip of the ureteral catheter was removed from the ureteroileal intersection, massive bleeding from the internal opening of the ureteral catheter was observed. We finally diagnosed her with ureteroiliac artery fistula and immediately requested the placement of a covered stent by endovascular treatment during interventional radiology. Angiography showed narrowing of the external iliac artery but did not detect a fistula to the urinary tract (Fig. 3a). A heparin-bonded stent graft (Gore Viabahn; W.L. Gore & Associates, Inc., Newark, DE, USA) was placed in the narrowed external iliac artery, followed by expansion of the stent graft with crimping (Fig. 3b). Although the patient’s gross hematuria disappeared thereafter, and no further episodes of hemorrhage occurred, she died 11 months later of progression of her primary disease.

## Discussion and conclusions

Although the onset of MUO is a sign of poor prognosis, improved anticancer chemotherapy has prolonged survival even in patients with advanced cancer who have MUO. Thus, a more active intervention for MUO is recommended. However, there are various disadvantages associated with urinary tract obstruction relief for MUO by polymer ureteral stent or nephrostomy, and treatment strategies have to be carefully decided to avoid unnecessary interventions, considering the prognosis. The full-length metallic Resonance stent is the first metal ureteral stent available for use in Japan, having been approved in 2014, and this breakthrough device may complement the shortcomings of conventionally used polymer stents. First, because the metal stent has a strong resistance to compression from outside of the ureter [6], stent failure is unlikely to occur despite strong compression of the ureter due to the progression of the primary disease. According to various reports, the incidence of stent failure using the conventional polymer stent is approximately 25–40% [10], whereas that of the metal stent is about 7–35% [11–15]. Second, because the median survival time of patients with MUO is less than 10 months, conventional polymer stents need 1.9 stent replacements prior to death [3], whereas metallic stents, with a maximum indwelling period of 12 months, may not require replacement. Hence, an increasing number of patients with MUO are being treated with metallic stents, and many reports have described their effectiveness and safety [16–21].

Ureteroarterial fistula with an indwelling ureteral stent is an uncommon complication, although this condition can be life-threatening, owing to potential massive blood loss. The possible greater incidence of this severe event could also have resulted from the use of ureteral stenting (double-J stents), higher radiation doses, underlying vascular disease, and a greater incidence of previous pelvic vascular and oncologic surgeries combined with longer general survival [22, 23]. The pathophysiology behind the development of ureteroarterial fistulas remains uncertain. Batter *et al.* suggested that pressure necrosis from chronic indwelling ureteral catheters could further contribute to the degeneration of the ureteral wall, leading to fistula formation [23]. Moreover, Krambeck *et al*. considered that disruption of the vasa vasorum leads to changes in the media and adventitia of the large arteries, and the ureters are fixed to the iliac vessels by developing inflammation and extensive fibrosis. When a ureteral catheter is placed, it acts as a counterbrace, leading to alterations in ureteral elasticity by transmitting the systolic arterial wave onto the ureteral wall and eventual necrosis of the ureteral and arterial wall ensues with subsequent fistula development [24]. In patients who underwent radiation therapy, the average time between radiotherapy and the onset of ureteroarterial fistula was 36 months, whereas the average time between stenting and onset of ureteroarterial fistula was 18 months [25]. Ureteral stenting therefore greatly contributes to the formation of ureteroarterial fistulas.

Early diagnosis of ureteroarterial fistula can reduce the mortality rate [26]; however, accurate diagnosis is difficult. Various radiological modalities, such as contrast-enhanced CT, arteriography, and retrograde urography, have low sensitivities of 38–50%, 25–50%, and 45–60%, respectively [23, 24, 27–30]. Therefore, once a ureteroarterial fistula is suspected or detected, multidisciplinary therapy must be promptly provided by specialists, including urologists, radiologists, and vascular surgeons. Although there is no standard treatment, endovascular treatment by percutaneous interventions has recently become the treatment of choice [31].

**Fig. 4:**
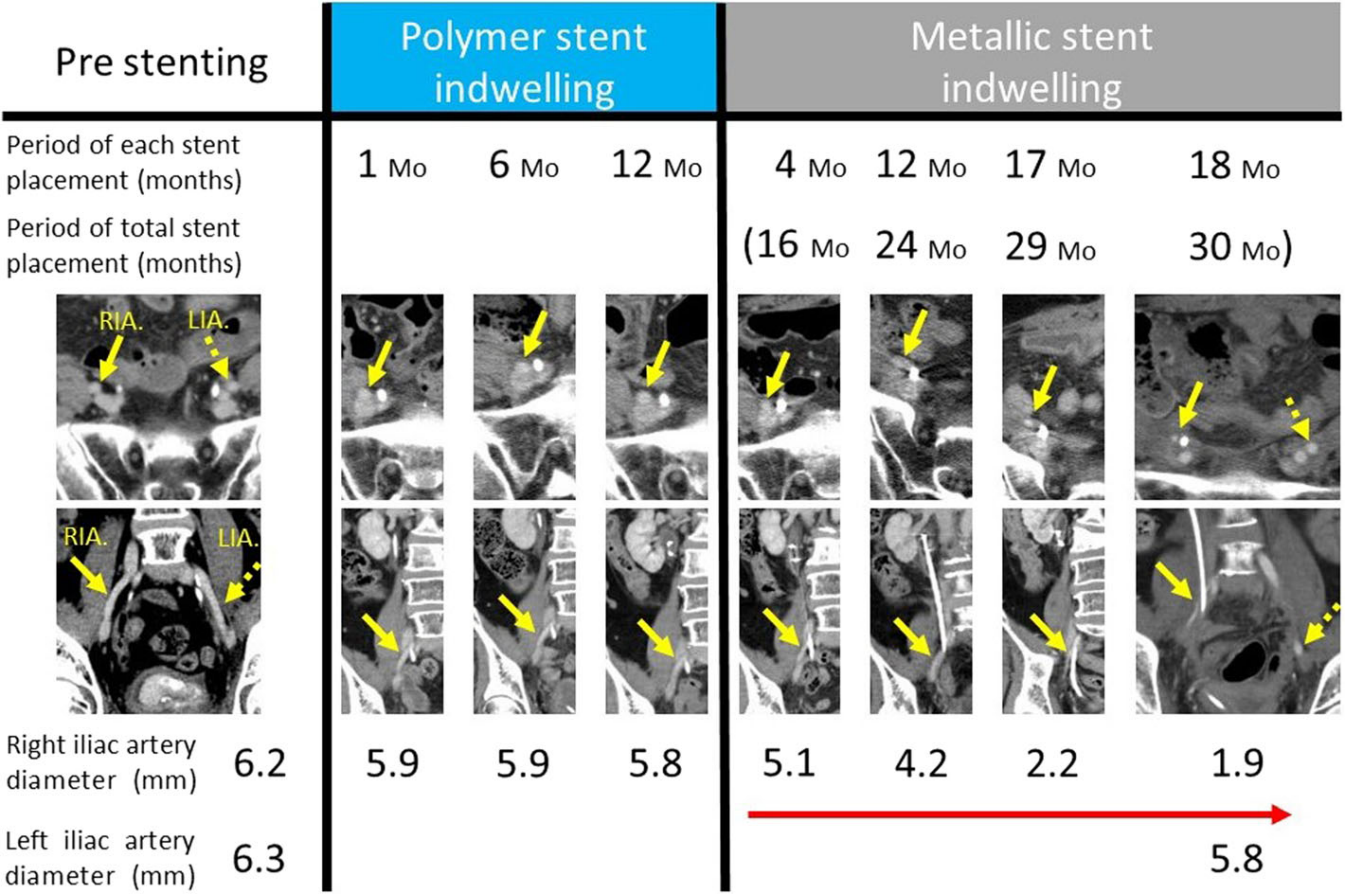
Details of the changes in diameter comparison between right and left iliac arteries. Right iliac artery narrows over time, especially during metallic stent indwelling (*yellow arrow*), whereas left iliac artery diameter did not change (*yellow dotted arrow*)

We present the first case of ureteroiliac artery fistula caused by a full-length metallic ureteral stent in a patient with MUO. Because our patient had many risk factors that could cause ureteroarterial fistula, such as previous abdominal oncologic surgeries, high-dose radiation therapy, and frozen pelvis in a female patient with metastatic cancer, as well as a long-term indwelling ureteral stent, we could immediately suspect ureteroarterial fistula and proceed to treatment, even for such a rare event. To verify if we could detect any signs of ureteroarterial fistula, we focused on the stenosis of the external iliac artery around the ureteral intersection as revealed by angiography. Data obtained by CT over time showed that the right external iliac artery diameter, 6.2 mm before stenting, remained almost unchanged at 5.8 mm during the first 12 months with the polymer stent placement. After replacement with a metallic stent, the diameter of the right external iliac artery narrowed increasingly to 4.2 mm at 12 months after placement and to 1.9 mm at 18 months of onset of ureteroarterial fistula (Fig. 4). CT showed no calcification of the arterial wall; thus, it was not a narrowing of the external iliac artery due to atherosclerotic changes. Metallic stents are more resistant to compression from external forces, although alternatively, they may also exert greater pressure on the extraureteral tissue, which may lead to chronic progressive iliac arterial narrowing and necrosis with fistula of the ureter.

The prognosis of patients with MUO depends on the cancer type, although, considering that gynecological cancers may have a better prognosis than gastrointestinal cancers [4], the timing of metallic stent placement might have been early in our patient’s case. However, it is also difficult to accurately predict the prognosis of advanced cancers, and it is necessary to carefully determine the indication for MUO relief. Because patients with MUO have a generally short life expectancy, they may not be able to survive until the onset of ureteroarterial fistula caused by metallic stents. Long-term placement of metallic stents in long-term survivors may have revealed the risk of developing ureteroarterial fistula. Without surgical intervention, long-term indwelling metallic ureteral stents for ureteral stenosis due to a benign disease, such as with indiscriminately permanent stent placement, may not be truly safe.

The metallic stent is a useful device that can maintain renal function without compromising quality of life of patients with MUO with a short life expectancy. However, it may exert higher pressure on the extraureteral tissue than conventional polymer stents due to its properties and may lead to development of ureteroarterial fistula if the stents are noticeable at the intersection with the iliac arteries. Ureteroarterial fistula should be suspected immediately if massive gross hematuria is observed in patients with indwelling ureteral stents. The external iliac artery diameter observed by CT may be helpful for predicting ureteroarterial fistula.

## Data Availability

Not applicable.

## Abbreviations

CT: Computed tomography
MUO: Malignant ureteral obstruction
